# Impact of COVID-19 Pandemic in a Brazilian High-Volume Aortic Center

**DOI:** 10.21470/1678-9741-2020-0567

**Published:** 2021

**Authors:** Ricardo R. Dias, José Augusto Duncan Santiago, Vagner Madrini Junior, Charles Mady, Fabio B. Jatene

**Affiliations:** 1 Department of Cardiovascular Surgery, Instituto do Coração (InCor), Faculdade de Medicina da Universidade de São Paulo, São Paulo, São Paulo, Brazil.; 2 Department of Cardiomyopathies and Aortic Diseases, Instituto do Coração (InCor), Faculdade de Medicina da Universidade de São Paulo, São Paulo, São Paulo, Brazil.

**Keywords:** COVID-19, Pandemics, Elective Surgical Procedures, Aortic Diseases, Mortality, Coronavirus Infections

## Abstract

**Introduction:**

The coronavirus disease 2019 (COVID-19) pandemic brought an unprecedented lack of control of what was to come. The intent of this document is to provide a balance of how much was ceased to be done for patients with aortic disease, to assess the mortality of these patients, and to show what happened to those who became COVID-19 positive during their hospitalization.

**Methods:**

From April 1st to July 31st 2020, the worst period of the pandemic in São Paulo, Brazil, the Institute’s aortic surgical patients operated on were evaluated and those were compared with patients operated during the same period in 2019.

**Results:**

In 2019, 88 surgeries were performed; most of them were elective (66 [75%]), 10 were urgent, and 12 were emergency surgeries. In 2020, during the COVID-19 pandemic, we operated on only 31 patients, being 74.2% non-elective surgeries (*P*<0,001). There was a higher mortality for patients operated on during the pandemic surge of COVID-19 (*P*<0,001), but it was not specifically related to infected patients.

**Conclusion:**

The COVID-19 pandemic had an impact on surgical volume and outcome of patients with aortic disease, although it did not directly increase mortality.

**Table:** 

Abbreviations, acronyms & symbols	
**AAA**	**= Abdominal aortic aneurysm**
**Arch A**	**= Arch aneurysm **
**Ac tA diss**	**= Acute type A aortic dissection**
**Ac tB diss**	**= Acute type B aortic dissection**
**Asc Ao A**	**= Ascending aortic aneurysm**
**Chron tA diss**	**= Chronic type A aortic dissection**
**Chron tB diss**	**= Chronic type B aortic dissection**
**COVID-19**	**= Coronavirus disease 2019**
**Desc Ao A**	**= Descending aortic aneurysm**
**SARS-CoV-2**	**= Severe acute respiratory syndrome coronavirus 2**
**TAAA**	**= Thoracoabdominal aortic aneurysm**

## INTRODUCTION

Categorical data were expressed as counts and percentages, and X^2^ test was used to analyze differences between groups. Statistical analyses were performed using IBM Corp. Released 2015, IBM SPSS Statistics for Windows, Version 23, Armonk, NY: IBM Corp., with significance at an alpha level of 0.05.

## RESULTS

In 2019, a total of 88 surgeries were performed; most of them were elective (66 [75%]), 10 were urgent, and 12 were emergency surgeries. In 2020, during the COVID-19 pandemic, we operated on only 31 patients, being 74.2% non-elective surgeries (*P*<0.001). When comparing the procedures of the two years, it is possible to observe the different clinical patient presentation, as illustrated in Table 1.

**Table 1:** Total number of surgeries from April 1^st^ to July 31^st^ (2019 and 2020), specifying its elective, urgent, and emergency character and its variation between the two periods.

	Total Surgeries - 2019 (N=88)	%	Total Surgeries - 2020 (N=31)	%	*P*-value
Elective	66	75%	8	25.8%	0.001
Urgent	60	11.4%	11	35.5%	0.004
Emergency	12	13.6%	12	38.7%	0.004
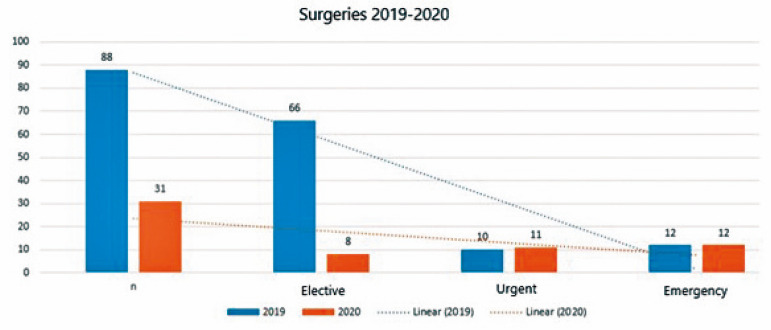					

**Fig. 1:**
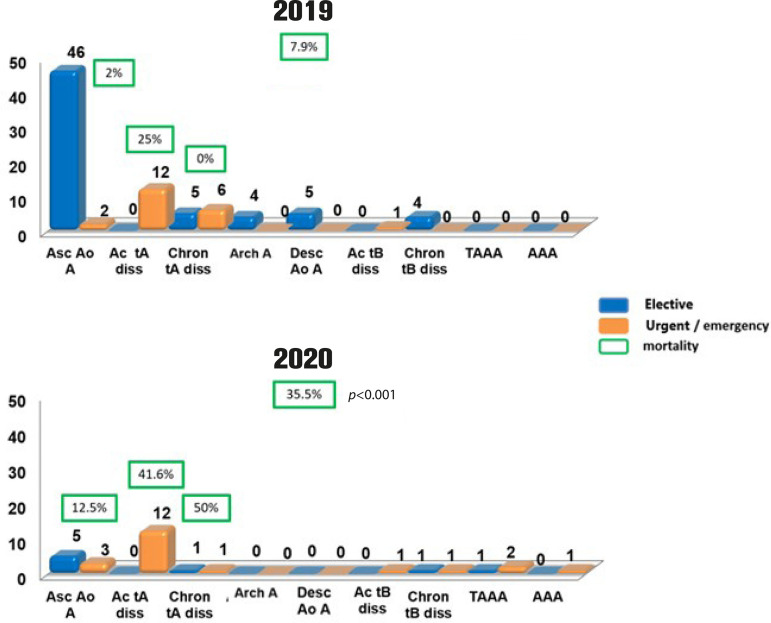
Number of patients operated on based on their diagnosis of aortic disease. In green, the general and specific hospital mortality during the studied period, in the three most common performed operations. AAA=abdominal aortic aneurysm; Arch A=arch aneurysm; Ac tA diss=acute type A aortic dissection; Ac tB diss=acute type B aortic dissection; Asc Ao A=ascending aortic aneurysm; Chron tA diss=chronic type A aortic dissection; Chron tB diss=chronic type B aortic dissection; Desc Ao A=descending aortic aneurysm; TAAA=thoracoabdominal aortic aneurysm

Most of the patients operated on in 2019 were those with ascending aortic aneurysm, with mortality rate of 2% (one in 48 patients). In 2019, there were two operations on an emergency basis due to aortic rupture *vs*. three in 2020 (4.2% urgent/emergency surgeries *vs*. 37.5%), but with no significant difference in mortality (2% *vs*. 12.5%; *P*=0.142) (Figure 1).

For type A aortic dissection, a different scenario is shown; the same incidence of acute cases, but with no difference in mortality (25% *vs*. 41.6%; *P*=0.059). For chronic type A aortic dissection, there was a significant decrease in the number of cases and an increase in the mortality rate (0% *vs*. 50%; *P*=0.015). For other aortic diseases or different affected segments, in general, no significant difference was observed, neither in incidence nor in mortality, mainly due to the small number of operated cases, as shown in Figure 1.

When analyzing overall mortality during the COVID-19 pandemic period and comparing with the same period in 2019, the difference was evident (7.9% × 35.5%; *P*<0.001) (Figure 1).

Table 2 presents the total number of operated cases during the study period in 2020, the hospital mortality, cases with COVID-19 (those in which the infection was brought from community or acquired during hospitalization), and the absence of difference in mortality rate among patients with and without COVID-19.

**Table 2:** 2020 total surgeries and hospital mortality, total coronavirus disease 2019 (COVID-19) cases, and mortality, in the three different clinical presentations for surgery.

	Total Surgeries - 2020	Total Mortality	%	COVID-19 Cases	COVID-19 (%)/Total	COVID-19 Mortality	%
N	31	11	35%	7	23%	2	29%
Elective	8	2	25%	2	25%	1	50%
Urgent	11	4	36%	3	27%	1	33%
Emergency	12	5	42%	2	17%	0	0%
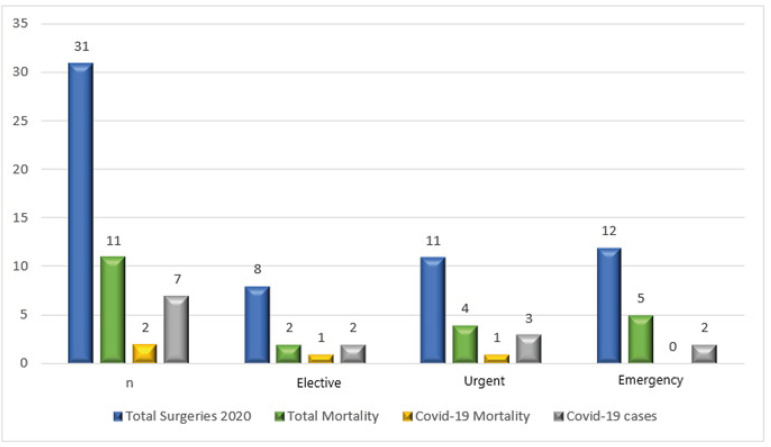							

Seven patients with known COVID-19 infection were operated on and two of them died (28.6%). Again, no difference in mortality was observed between patients with and without COVID-19 operated on during the study period (Table 2).

## DISCUSSION

The impact of COVID-19 pandemic surge has been felt differently across the cardiovascular community at national and regional levels due to differences in prevalence of infection rates, social characteristics, and health care system response^[5]^.

Academic centers in São Paulo comprised the majority of institutions with high burden of COVID-19 patients, consuming the limited resources and personnel of our Institutes in their daily practice. The Hospital das Clínicas, Faculdade de Medicina da Universidade de São Paulo, acted as the referral Institute for all other centers in the State, dictating the normatives for care of patients of the public health care system. In this scenario, the Instituto do Coração redirected its resources to other medical specialties of the general building to assist their patients, and stopped assisting non-urgent cardiovascular patients.

In this new reality, the Institute’s patient presentation changed completely. For months, our high-volume Cardiovascular Surgery Institute changed its practice, limiting the elective procedures to high-priority surgeries. Consequently, the Aortic Disease Unit experienced a significantly decrease in procedures, an increased in morbidity and mortality of its patients, mainly due to the momentary change in hospital characteristics of medical assistance, the mixture of specialties and conducts, and the impact of the pandemic on patients and health professionals responsible for their care.

The difference in the clinical presentation of the operated patients was evident, as was the increase in mortality. It is important to highlight that, despite the patients’ fear of coming to the hospital for care, the non-elective cases did not decrease. On the other hand, elective surgeries were suspended by the executive branch of the State.

Differently from what was observed for other surgical groups in our Institutes, it can be said that the COVID-19 infection did not directly impacted in our surgical mortality, as it has been shown. But the pandemic itself, the catastrophic scenario of the health system, impacting medical and paramedical assistance, the reprioritization of wards and intensive care units for COVID-19 treatment, the transitional institutional changes to general assistance, the limited workforce, and the fear of the virus’s presence clearly played a crucial role in worsening surgical outcomes, although it was not measured.

It is foreseeable that reduction in aortic surgery capacity will impact for several months or longer, but there is no time for tears or regrets and, as proposed by dr. Vervoort et al.^[6]^, now it is time for collaboration, rather than fragmentation, time to provide the necessary care for our patients, while bettering our understanding of the complexities brought upon individuals’ cardiovascular health due to COVID-19^[6]^.

Reprioritization of some patients will be necessary given the dynamic nature of cardiovascular diseases. It is time to embrace our patients, to protect the most vulnerable, to deal with health disparities, to work ethically, with attention to ethnic and gender disparities, to work well, and to quickly reduce the waiting list for surgeries, which tripled during this dark period^[7,8]^.

The recovery of the previous year’s surgical outcomes requires multisectoral and collaborative efforts to ensure the safety of our patients.

## Conclusion

The COVID-19 pandemic had an impact on surgical volume and outcome for patients with aortic disease, although it did not directly increase mortality.

**Table:** 

Authors' roles & responsibilities	
RRD	Substantial contributions to the conception or design of the work; or the acquisition, analysis, or interpretation of data for the work; drafting the work or revising it critically for important intellectual content; final approval of the version to be published
JADS	Substantial contributions to the conception or design of the work; or the acquisition, analysis, or interpretation of data for the work; drafting the work or revising it critically for important intellectual content; final approval of the version to be published
VMJ	Substantial contributions to the conception or design of the work; or the acquisition, analysis, or interpretation of data for the work; drafting the work or revising it critically for important intellectual content; final approval of the version to be published
CM	Substantial contributions to the conception or design of the work; or the acquisition, analysis, or interpretation of data for the work; drafting the work or revising it critically for important intellectual content; final approval of the version to be published
FBJ	Substantial contributions to the conception or design of the work; or the acquisition, analysis, or interpretation of data for the work; drafting the work or revising it critically for important intellectual content; final approval of the version to be published
